# Does Scanner Choice Matter for the Design of Foot Orthosis?

**DOI:** 10.3390/s25030869

**Published:** 2025-01-31

**Authors:** Komal Chhikara, Sinduja Suresh, Scott Morrison, Dean Hartley, Kerrie Evans, Marie-Luise Wille, Müge Belek Fialho Teixeira, Bridget Hughes, Natalie Haskell, Amanda Beatson, Marianella Chamorro-Koc, Judith Paige Little

**Affiliations:** 1 School of Mechanical, Medical and Process Engineering, Faculty of Engineering, Queensland University of Technology, Brisbane, QLD 4000, Australia; s.suresh@qut.edu.au (S.S.); m.wille@qut.edu.au (M.-L.W.); j2.little@qut.edu.au (J.P.L.); 2 Centre for Biomedical Technologies, Faculty of Engineering, Queensland University of Technology, Brisbane, QLD 4000, Australia; 3 ARC Training Centre for Multiscale 3D Imaging, Modelling, and Manufacturing, Queensland University of Technology, Brisbane, QLD 4000, Australia; 4 Biomechanics & Spine Research Group at the Centre for Children’s Health Research, Faculty of Engineering, Queensland University of Technology, Brisbane, QLD 4000, Australia; 5 iOrthotics, Brisbane, QLD 4030, Australia; scott@iorthotics.com.au (S.M.); dean.hartley@healthia.com.au (D.H.); 6 Healthia Limited, Brisbane, QLD 4006, Australia; kerrie.evans@healthia.com.au; 7 Faculty of Medicine and Health, School of Health Sciences, The University of Sydney, Sydney, NSW 2006, Australia; 8 School of Architecture and Built Environment, Faculty of Engineering, Queensland University of Technology, Brisbane, QLD 4000, Australia; muge.teixeira@qut.edu.au; 9 School of Education, Faculty of Creative Industries, Education, and Social Justice, Queensland University of Technology, Brisbane, QLD 4000, Australia; by.hughes@qut.edu.au; 10 School of Design, Faculty of Creative Industries, Education and Social Justice, Queensland University of Technology, Brisbane, QLD 4000, Australia; n.haskell@griffith.edu.au (N.H.); m.chamorro@qut.edu.au (M.C.-K.); 11 School of Architecture, Industrial Design and Planning, Griffith University, 1 Parklands Dr, Southport, QLD 4215, Australia; 12 QUT Business School, Centre for Decent Work and Industry (CDWI), Queensland University of Technology, Brisbane, QLD 4000, Australia; 13 QUT Design Lab, Faculty of Creative Industries, Education and Social Justice, Queensland University of Technology, Brisbane, QLD 4059, Australia

**Keywords:** foot, orthoses, three-dimensional, geometry, scanning, orthotic design

## Abstract

A variety of 3D volumetric scanners and smart-device applications are currently being used in podiatry for recording virtual foot data. The accuracy and reliability of these devices vary, resulting in a large variation in the quality of foot scans used for orthotic design. While it is widely believed that a higher quality scanner yields a better scan and thus is expected to produce a more accurate orthotic design, the direct impact of scanning quality on orthotic design has not yet been tested. Therefore, in this study, three commonly used industrial 3D scanners with varying output qualities were used to obtain foot scans of three participants in two weight-bearing conditions. A total of 54 foot scans were obtained, out of which 18 were used to design orthotic insoles using commercial software (FitFoot360). We found variation in the quality of foot scans produced by the different scanners (61.75 ± 2.23% similarity of the foot scans showing a deviation of less than ±1 mm). However, there were no significant differences in the designed foot orthoses within the same weight-bearing condition (83.59 ± 1.97% similarity of the orthotic designs showing a deviation of less than ±1 mm). The medial arch height and heel width differed significantly only when the weight-bearing condition was changed. The findings from this study suggest that the industrial design and production of an orthotic insole using current methods does not depend on the scanning quality of the scanner used but is dependent on the extent of weight bearing.

## 1. Introduction

Orthotic devices are used to provide external support and improve pain and function [1]. Foot orthoses generally aim to prevent deformity along with accommodating and supporting the foot of individuals with pathologies [2]. Foot orthotic management is usually prescribed by podiatrists/orthotists and can be either prefabricated orthoses (off the shelf) or custom-fabricated orthoses [3,4]. Custom orthoses can be produced with conventional manufacturing methods (casting) and/or advanced manufacturing methods. Advanced manufacturing methods use 3D scanning and 3D printing technologies for orthotic production. The production workflow involves scanning the foot, processing the scanned image, and digitally designing the orthoses in 3D through appropriate software and production through additive manufacturing and fitment checks [5,6].

Customisation of the orthotic design is achieved by recording measurements of the foot and replicating the foot geometry either with casting or 3D scanning methods [7]. The traditional casting method requires expertise to accurately mould the plaster of Paris (PoP) bandages to the foot whilst maintaining correct foot and ankle alignment and shaping the medial longitudinal arch (MLA) and Achilles tendon [8]. To reduce the variability associated with traditional casting methods, and with advancements in technology, 3D scanning methods are being used more frequently in clinical practice. Three-dimensional scanners are comparatively faster and are more accurate in capturing the complex geometry of the foot compared to traditional casting methods [9,10]. Additionally, changes can be made to a digital design without needing to re-cast the foot, and digital scans are easy to share between clinics and manufacturing laboratories, eliminating the need for storing casts and positive PoP moulds [11]. Studies demonstrate that 3D surface scanners have greater accuracy, robustness, and reliability than traditional methods and can precisely capture detailed foot anthropometric data [12,13]. Performing a 3D scan to capture foot geometry is not only more efficient but is a more comfortable method for the patient compared to traditional casting methods [13,14,15]. When performing a scan, the position of the patient’s foot can depend on the patient’s presentation and preference, the podiatrist/orthotist’s clinical practice or habits and the availability of equipment.

The podiatry industry is gradually inclining towards the adoption of digital 3D modelling techniques fuelled by the increasing availability and improving accuracy of digital scanning equipment [7,11,16,17,18,19,20]. There are various 3D surface scanners that are currently used in the industry to capture the surface topography (ST) of feet. Some 3D scanners use structured light for capturing the 3D anthropometric data [21,22,23] such as the Artec range of scanners (Artec Group, Senningerberg, Luxembourg) while other scanners use infrared light or depth sensors to capture ST [13,24].

Several studies have compared these 3D scanners with the traditional method of capturing foot and ankle morphology. In one of the studies, Farhan et al. [25] investigated the accuracy and speed of Artec Eva and Structure Sensor II (SS) in comparison to the plaster cast measures and found that all the approaches had similar accuracy in capturing lower leg morphology for the fabrication of ankle–foot orthosis (AFO) in a non-weight-bearing (NWB) position. Powers et al. [11] compared measurements obtained from SS with manual measurements taken in a half-weight-bearing position (HWB). They reported that SS-based scanners are valid and reliable for capturing lower limb morphology for the fabrication of AFO. Carroll et al. [7] performed a similar comparison by comparing measurements between plaster casts and digital scans taken by a white light scanner in the NWB position. They also reported that digital scanning was a reliable technique as all the measurement comparisons demonstrated less variability between measurements.

On the other hand, some studies have reported the accuracy and reliability of one scanner over the other in terms of capturing the morphology of human body parts [23,26,27]. A review study conducted by Silva et al. [28] discussed the emerging use of 3D scanners in producing custom orthotics. They reported in the review that photogrammetry and structure light scanners were the most suitable scanners to scan the human body. A study conducted by Redaelli et al. [10] comparing several low-cost devices and high-resolution sensors for the fabrication of orthotic devices by scanning parts of a mannequin suggested that the SS scanner had comparable accuracy with the high-resolution scanners. A different study conducted by Farhan et al. [29] compared the accuracy and speed of seven industrial scanners for capturing foot and ankle morphology. They reported that three out of the seven scanners captured more accurately and faster than other scanners, suggesting that the 3D scanners which did not capture sufficiently should be avoided in the clinics.

Despite the abundance of studies in the literature on the accuracy and reliability of various surface scanners in podiatry, the focus has solely been on measuring the quality of the foot scans obtained by comparing measurements of the foot taken from these scans [10,11,13,19,30]. This is because it is widely believed that the design and success of orthotic fitment depends on obtaining a good quality foot scan that sufficiently and accurately captures the surface anatomy of the patient’s foot. However, it is still unknown if a difference in the quality of a foot scan impacts the final orthotic design. To our knowledge, there are no studies that have investigated whether scanner type influences the orthotics produced. This study therefore aims to compare the differences in foot orthoses designed using the foot scans obtained in two different positions using three common 3D topographic scanners and applications used by the industry experts.

## 2. Materials and Methods

**Figure 1:**
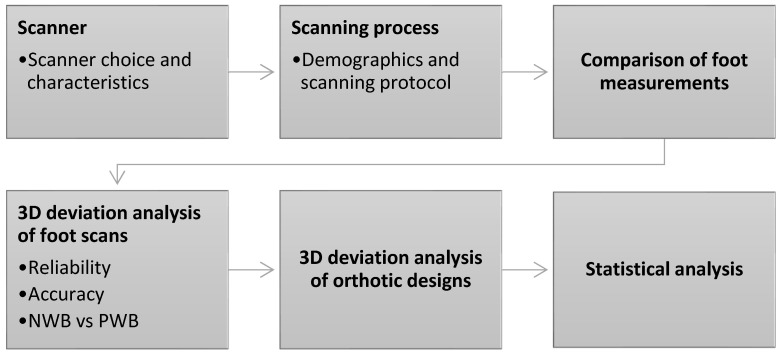
Flowchart for the methodological process.

The methodological process is illustrated in Figure 1.

This study included three scanners commonly used in podiatry clinics in Australia:

1. Artec Leo white light scanner (Artec Group, Senningerberg, Luxembourg), a structure light scanner suitable for applications requiring high-precision and detailed scanning [31,32].
2. iOrthotics laser flatbed scanner (iOrthotics, Brisbane, Australia), a laser scanner that is suitable for applications that require the foot to be maintained in a specific corrected position depending on the pathology [16,33].
3. iOrthotics iOS smart-device application using Truedepth phone camera, a phone-based application scanner suitable for applications where high precision may not be critical and the aim is portability and ease of use [34,35].

These scanners represented a wide variety of scanning technologies including high-resolution, laser scanning, and phone app-based methods, ensuring comprehensive evaluation. Technical details of the scanners used in this study are described in Table 1.

Three healthy individuals, two males and one female with an average age of 32.66 ± 2.88 years, average weight of 63.33 ± 15.27 kgs, and height of 165.66 ± 17.09 cm, were recruited for this study. The participants exhibited no pathological deformities or abnormalities in the foot. Three anatomical landmarks (ALs) on the plantar foot region were marked using palpation, the centre of the heel (AL 1), the first metatarsal (AL 2), and the fifth metatarsal (AL 3), before scanning the participant to facilitate controlled landmark-based alignment between scans and landmark-based orthotic designing [36,37,38,39,40,41].

**Figure 2:**
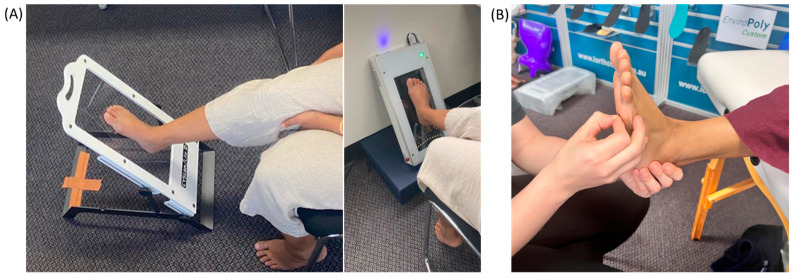
(**A**) PWB: the picture on the left shows the scanning position with Perspex glass and the picture on the right shows the PWB position on the laser flatbed scanner; (**B**) the NWB position for scanning (3D stickers being placed on anatomical landmarks).

The right foot of the participants was scanned in two positions commonly used in clinical practice. The positions were selected to assess the impact of different weight-bearing positions on the foot scans and orthotic designs. In the first position, the participants were seated on a chair with their feet placed on a piece of Perspex glass angled at 45 degrees. This partial weight-bearing (PWB) position is illustrated in Figure 2. The iOrthotics laser flatbed scanner has a built-in glass platform which was inclined to 45 degrees to mimic the Perspex glass. The angle of the hip and knee were measured using a magnetic angle finder during the scanning to standardise the participant’s position. The angles were kept consistent to maintain the shin’s position at 90 degrees with respect to the Perspex glass. For the NWB position, the participant sat on a portable treatment table with the right foot extended beyond the edge of the bed (Figure 2B).

In the NWB and PWB positions, three scans using each of the three scanners in succession, i.e., a total of nine scans in each position, were obtained for each participant. For foot scans, the reliability of the scanners was assessed using 27 scans from the three participants and the accuracy of the scanners was assessed using 18 scans from the three participants for robust statistical analysis calculated with 80% power and a 95% confidence interval. The participants were asked to remain still to reduce the impact of movement artefacts. The scans obtained from the flatbed scanner and smart-device application were directly exported as meshes, whereas the Artec raw scans were stitched on the associated software Artec 17 professional (Artec Group, Senningerberg, Luxembourg) to obtain fused watertight meshes before further processing.

### 2.1. Comparison of Foot Measurements

To measure the foot fast and reliably, a semi-automated measurement tool that allowed for parametric user input was developed on the 3D modelling and graphical programming software Rhino 7 with the Grasshopper add-on (Robert McNeel and Associates, Seattle, WA, USA). The 3D mesh model can be manipulated on Rhino while user inputs to the algorithm are accepted through the Grasshopper interface.

**Figure 3:**
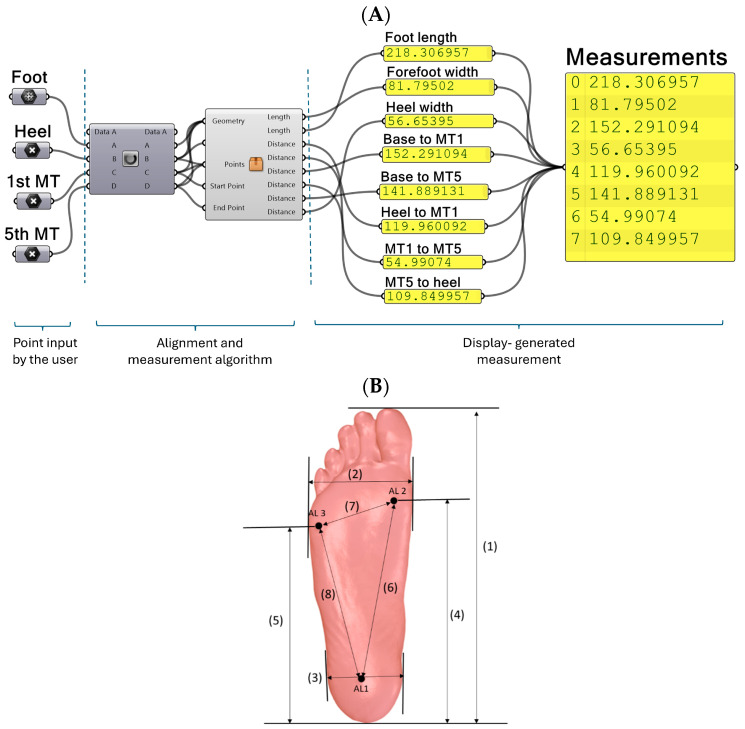
(**A**) Developed RG workflow. (**B**) Measurements taken in the plantar region: (1) foot length, (2) forefoot width, (3) heel width, (4) base to first MT head (5) base to fifth MT head, (6) heel to MT1, (7) MT1 to MT5, and (8) MT5 to heel.

In Rhino–Grasshopper, the scans were aligned to the world coordinate system using the three plantar landmarks designating the heel landmark as the origin. The line passing through the centre of MT1 and MT5 landmarks was assigned as the y-axis. The developed algorithm then automatically calculated eight basic foot measurements in the foot. Figure 3 demonstrates the algorithm and the measurements performed on the plantar region of the foot scans for (1) foot length, (2) forefoot width, (3) heel width, (4) base to first MT head (5) base to fifth MT head, (6) heel to MT1, (7) MT1 to MT5, and (8) MT5 to heel.

### 2.2. Three-Dimensional Deviation Analysis of Foot Scans

For standardisation during 3D deviation analysis, the NWB and PWB scans were trimmed from the base plane. Since all scanners captured different heights of the foot from the plantar surface, the cut-off level was determined by the scanner that captured the least amount of information (scans obtained from the flatbed scanner) for all participants. These trimmed scans were then imported into GeoMagic Control X. Two models of each position were compared at a time, one designated as the reference and the other as the floating model for comparison. The maximum and minimum colourmap range was maintained at ±5 mm and the tolerance level was maintained at ±1 mm (this tolerance is considered standard for designing orthosis in the industry) [10].

Reliability: The three scans obtained from each scanner were compared with each other for intra-scanner reliability: scan 1 vs. scan 2, scan 2 vs. scan 3, and scan 3 vs. scan 1. This comparison was carried out for NWB and PWB foot scans from each scanner. The results were then averaged across the three sets of scans.

Accuracy: One scan out of the obtained three scans from each scanner was analysed for 3D deviation (Artec scan 1 vs. flatbed scan 1, flatbed scan 1 vs. Truedepth scan 1, and so on) for inter-scanner accuracy. The mean of each deviation analysis obtained for the 3 participants was calculated from the results obtained by each scanner comparison.

NWB vs. PWB: The NWB scan 1 was compared with the PWB scan 1 and was followed by a comparison for each set of NWB and PWB scans. This deviation analysis was repeated for each scanner.

### 2.3. Three-Dimensional Deviation Analysis of Orthotic Designs

Digital orthosis designs were made by one expert industry designer from iOrthotics (Healthia Limited, Bowen Hills, Australia) on the FitFoot360 version 3 (Fit360 Ltd., Worcestershire, UK) software using only the first scan from each scanner for each participant, both in the NWB and PWB positions. These 18 orthotic designs were compared for 3D variations.

### 2.4. Statistical Analysis

Data were analysed using SPSS Version 29.0 (SPSS Inc., Chicago, IL, USA). A one-way analysis of variance (ANOVA) was performed with post hoc Student–Newman–Keuls (SNK) tests. Error bars were used to indicate standard deviations. Significance levels were set at *p* < 0.05 (significant), *p*  <  0.01 (highly significant), *p*  <  0.001 (very highly significant), and *p*  <  0.0001 (extremely significant).

## 3. Results

**Figure 4:**
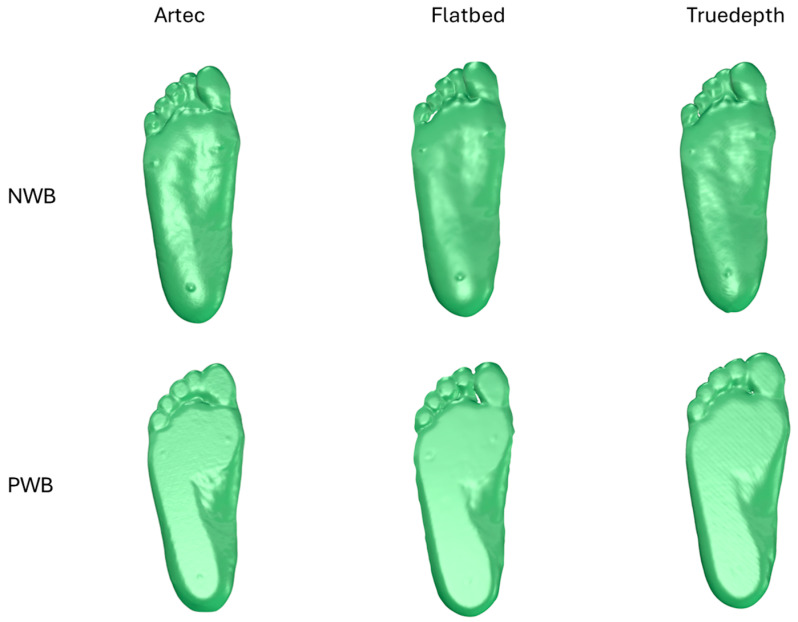
Picture of foot scans obtained from each scanner.

The data were analysed using GeoMagic Control X (version 2022, GeoMagic Inc., Rock Hill, SC, USA). Images of the scans obtained from each scanner are shown in Figure 4. All three scanner results showed details of the physical features of the foot. Only the Artec scanner captured the full arch height and covered the medial, lateral, and dorsal geometries. The other two scanners only captured the plantar region.

### 3.1. Comparison of Foot Measurement Results

**Figure 5:**
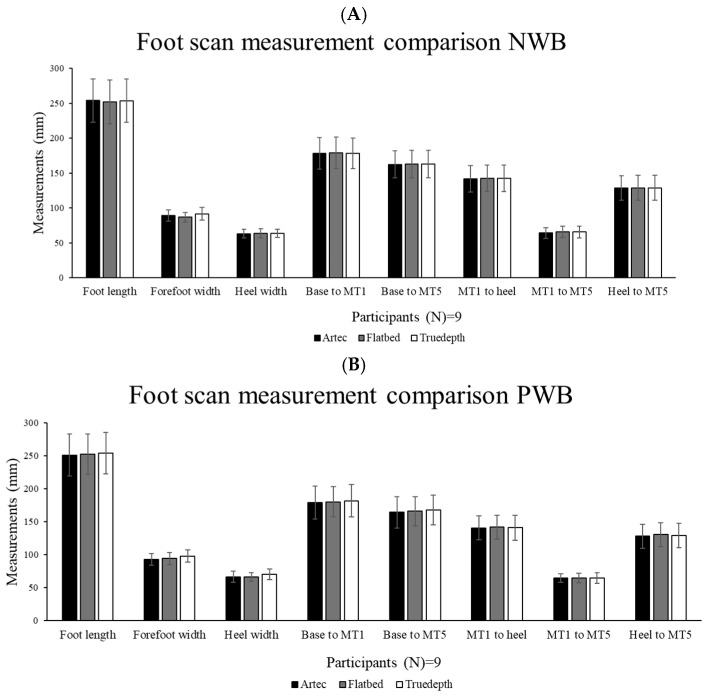
ANOVA and post hoc Student–Newman–Keuls (SNK) tests for (**A**) NWB and (**B**) PWB measurement comparison (N = 9). No significant differences were found.

The gross digital foot measurements showed non-significant differences between the three scan acquisition types (mean of three values) graphed in Figure 5.

### 3.2. Three-Dimensional Deviation Analysis of Foot Scans

**Figure 6:**
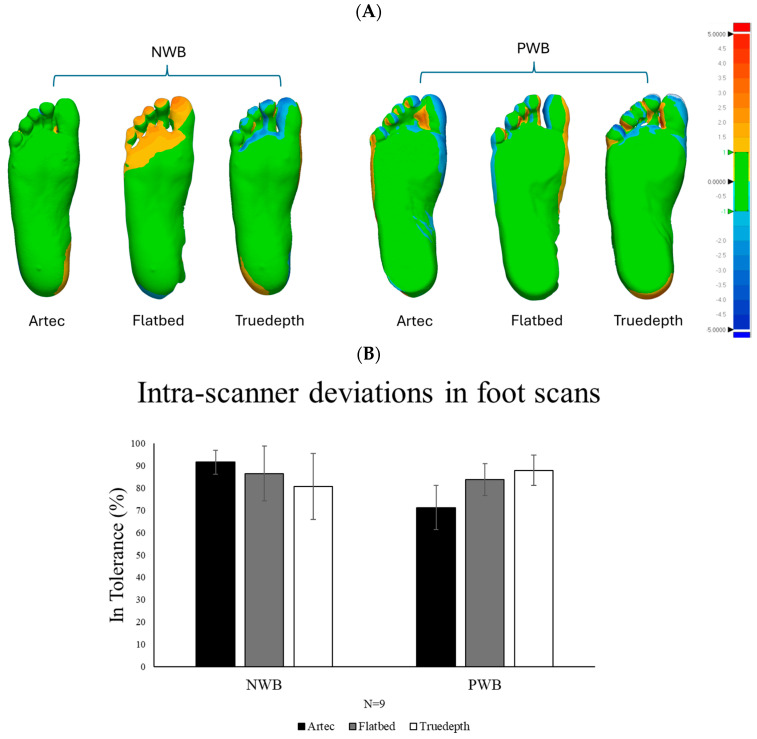
(**A**) Scanner reliability analysis for NWB and PWB foot scans (N = 9) (mean of scan measurements: 1 vs. 2, 2 vs. 3, and 3 vs. 1). Green region indicates acceptable deviation, also referred to as “in tolerance” in which the orthotic designs conform within a range of ±1 mm. The red colour indicates all the positive values exceeding +1 mm and the blue region indicates all the negative values beyond −1 mm. (**B**) % similarity in foot scans obtained from the same scanner.

Reliability: The intra-scanner reliability test shows results for the extent to which the repetitive foot scanning yielded the same results. As illustrated in Figure 6, Artec scans showed minimal 3D deviation in the toes. All other scanners showed high similarity in the scans with any 3D deviation within the tolerance level (denoted by the green colour). There were some variations observed in the toe region, especially in the NWB foot scans, likely due to uncontrolled toe movement between scans.

**Figure 7:**
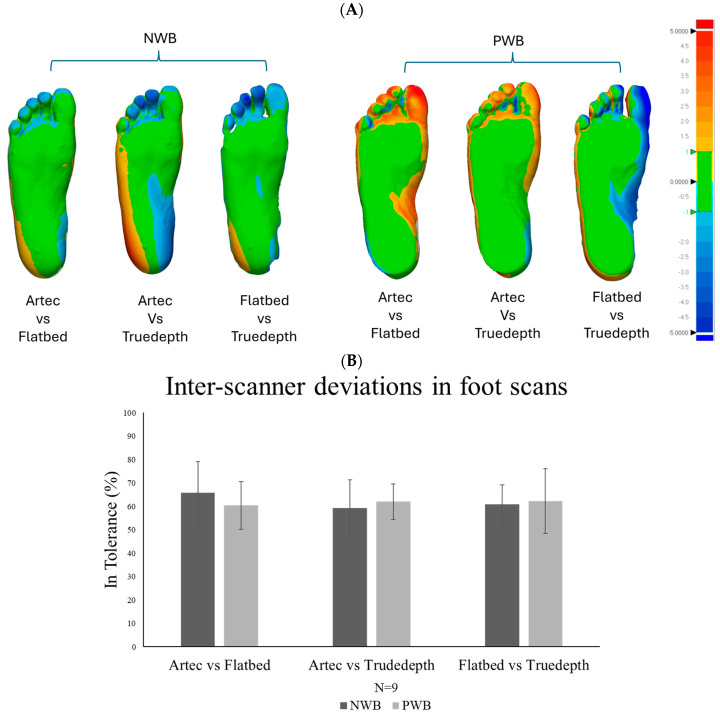
(**A**) Scanner accuracy analysis for NWB and PWB foot scans (N = 9). The green region indicates acceptable deviation, also referred to as “in tolerance” in which the orthotic designs conform within a range of ±1 mm. The red colour indicates all the positive values exceeding +1 mm and the blue region indicates all the negative values beyond −1 mm. (**B**) % similarity in foot scans obtained from different scanners.

Accuracy: The inter-scanner accuracy revealed differences around the toes, but most of the foot scans were within tolerance when compared to Artec scans. Figure 7 shows the variation graphs and images of the 3D comparison. As illustrated, all scans were within tolerance in the areas relevant for orthosis design (such as the arch height and the heel).

**Figure 8:**
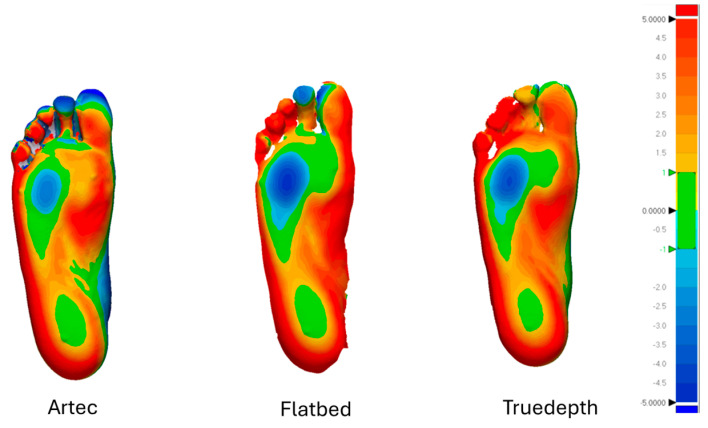
NWB vs. PWB foot scan analysis. The green region indicates acceptable deviation, also referred to as “in tolerance” in which the orthotic designs conform within a range of ±1 mm. The red colour indicates all the positive values exceeding +1 mm and the blue region indicates all the negative values beyond −1 mm.

NWB vs. PWB: The NWB vs. PWB inter-scanner 3D deviation analysis was performed separately for each scanner. All the 3D deviation analyses for NWB vs. PWB yielded similar results with the highest differences seen in the arch and hindfoot region as shown in Figure 8.

### 3.3. Three-Dimensional Deviation Analysis of Orthotic Designs

**Figure 9:**
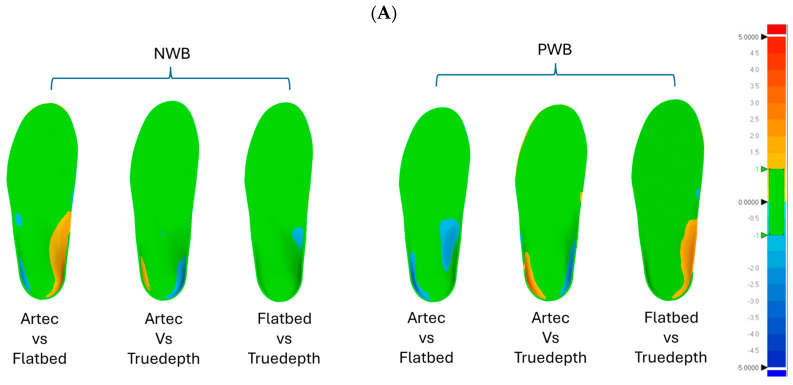

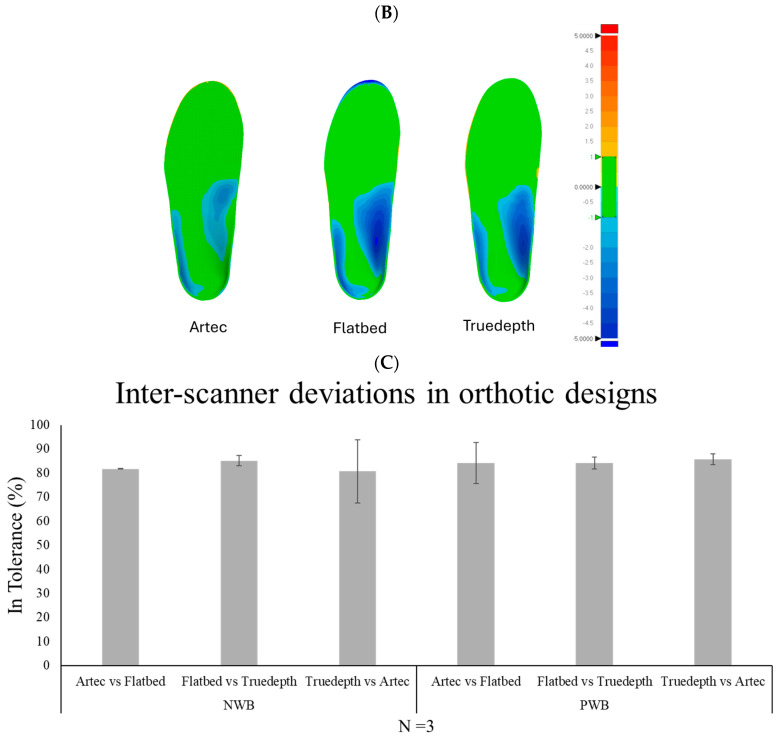
Orthotic design analysis for (**A**) NWB and PWB and (**B**) NWB vs. PWB orthotic designs (N = 3). The green region indicates acceptable deviation, also referred to as “in tolerance” in which the orthotic designs conform within a range of ±1 mm. The red colour indicates all the positive values exceeding +1 mm and the blue region indicates all the negative values beyond −1 mm. (**C**) % similarity in orthotic designs created for foot scans obtained from different scanners in NWB and PWB conditions.

The inter-scanner orthotic design comparison showed that there is a non-significant difference between the orthotic designs from every foot scan. The NWB vs. PWB orthotic designs showed significant deviations in the medial arch area. As illustrated in Figure 9, it could be seen in all the scanners.

## 4. Discussion

This study was conducted to compare the differences in foot orthoses designed using the foot scans obtained in two different positions using three 3D topographic scanners commonly used by the podiatry industry.

The scanning quality from each scanner showed that the toes could not clearly be distinguished in the foot scans taken using the Truedepth phone scanner. However, high-resolution toe capture is not critical for the design of orthotic insoles so this scanner may still be suitable for scanning purposes. The three anatomical landmarks on the plantar region of the foot were clearly visible in all the scans, which indicates that these scanners can capture small details. This is significant for the purpose of orthotic designing, particularly for people with diabetic foot disease which requires surface detail around lesions and sores for pressure offloading.

Variability in the gross measurements of the foot was statistically non-significant overall, suggesting that all three scanners, regardless of scanning quality, captured an overall similar foot morphology. The results obtained from comparing the foot measurements between successive scans from the same scanners validated the repeatability of the scanners. The reliability of each scanner was assessed by conducting multiple scans of the same participants under uniform conditions. Several studies [10,11,19,30,31,32,33,34,35,42,43] have compared different scanners (structure light, infrared, and laser) in terms of measurement accuracy and reliability. Powers et al. [11] and Rogati et al. [42], in their studies, reported that low-cost scanners can produce a reliable foot geometry (good to excellent ICC values = 0.84–0.99) for the purpose of orthotic designing. Stijn et al. [30] also reported good repeatability (root mean square error below 3 mm) after obtaining 3D scans of the plantar foot surface of 14 healthy subjects using a depth camera-based scanner. Similar results were seen in our study for the accuracy of the scanners, as all the scans were within the tolerance values. Minimal deviations (less than 5%) were observed in the forefoot region; however, they are generally not considered to be of high importance while designing foot orthoses [44,45] as the arch and heel are more important and harder to modify post-manufacture.

The 3D comparison between the orthotic designs revealed similarities in the 3D models for a large portion of the geometry. The inter-scanner orthotic design comparison showed no significant differences, indicating that the scanner type had little impact on the design. This result is contrary to the literature suggesting avoidance of certain scanners due to their low scanning quality which could possibly affect the orthotic designs [10,28,29]. However, the orthotic designs did vary between NWB and PWB input scans (Figure 8) and showed a minimum difference of approximately 5 mm in the medial longitudinal arch and the heel region of both positions (dark blue regions in Figure 9B). This difference could significantly impact the fitting of the orthosis on the foot, leading to increased post-processing like grinding of the orthotics, lower comfort, and possible complications for patients. It should be noted that the primary focus of the study was on the variation introduced by the scanning quality, and no notable inter-participant variation was observed in the results.

Currently, there appears to be no consistency in the input scans received in relation to scanner type, foot positioning, and degree of weight bearing. While it seems that scanner type is not a significant influencer in the design process, future research to improve standardisation in the other two areas focusing on foot position and weight bearing could significantly reduce the variability in the design process and result in fewer modifications after the manufacture and fitting processes.

Given that the choice of scanner does not significantly impact the design of orthotic insoles, it indicates that a low-cost scanner can be used in rural areas or resource-limited settings in the Australian population for the production of reliable orthotic insoles, making orthotic services more accessible. By studying and providing insights about the scanner choice on the orthotic design, this study aims to assist in the selection of the appropriate scanning technologies for different clinical and practical settings across Australia. The integration of 3D scanning technology in the field of podiatry/orthotics can present challenges for traditionally as well as digitally trained practitioners. Evidence-based knowledge could help guide clinicians and researchers to make appropriate decisions for the targeted population. However, several other factors must be considered such as ease of use, maintenance, portability, and ease of data transfer to the orthotic manufacturer. Full implementation in regional clinics will require a larger investment in the education and training of podiatrists/allied health professionals in rural regions to attain consistency in the scanning protocol [46,47].

This study had some limitations. The amount of weight put by the participants onto the Perspex glass scanning frame to simulate a stable and load-bearing position during PWB foot scans could not be quantified. In addition, no pathology was investigated as the primary focus of this study was scanning quality and the resulting orthotic insole, which can be used as control data in future studies since the results are independent of foot pathologies. Additionally, orthotics are designed differently for different types of foot pathologies; therefore, the criteria for reliability would be different. For example, common foot pathologies such as ulcers and lesions need foot orthotics to be designed based on offloading requirements. In this context, the scanner should be able to capture the necessary colour and texture information along with the high-resolution details around the ulcers and lesions allowing for correct demarcation of their limits. Future studies with pathological foot conditions could facilitate advanced decision making on scanning technology that can be appropriately used for different conditions.

## 5. Conclusions

One of the most common questions asked by podiatrists when transitioning from traditional manufacturing methods to digital volume capture and advanced fabrication methods is “Which scanner should I use?”. This study aimed to answer this question. Despite several reports by other studies on the need to choose a high-resolution scanner to achieve high-quality foot scans, we observed that the weight bearing of the foot plays a more important role in orthosis design than the choice of the scanner itself in the context of orthotic insole design for a healthy foot. However, this is only true when orthoses are designed with the current standard workflows of using gross measurements from a 3D scan to choose a pre-existing template from which an orthosis is designed. We expect that the choice of scanner, and therefore the accuracy of the foot scan, will become more important as design technology advances, and the degree and automation of customisation will be increased.

## Figures and Tables

**Table 1:** Scanner details.

Scanner	Manufacturer	Technology	Max Accuracy	Resolution	Software	Cost
Artec Leo	Artec 3D	White Light	0.1 mm	2.3 mp	3D	USD 34,800
iOrthotics Laser Flatbed	ScanPod3D	Laser	1.0 mm	1.0 mm	3D	USD 2000
TechMed Truedepth Phone Camera	Apple	Depth Camera	1.0 mm	12 mp	3dSizeMe	USD 780 per month

## Data Availability

The raw measurements, deviation analysis statistics, and statistical analysis used in the study will be made fully available to the public via QUT’s Institutional Research Data Finder (RDF) after the paper has been published. Through the RDF, non-institutional researchers may be granted access to these data after making a request to myself as the first author (Komal Chhikara, komal.chhikara@hdr.qut.edu.au) and owner of the RDF entries.
